# The Role of Endoplasmic Reticulum in the Differential Endurance against Redox Stress in Cortical and Spinal Astrocytes from the Newborn SOD1^G93A^ Mouse Model of Amyotrophic Lateral Sclerosis

**DOI:** 10.3390/antiox10091392

**Published:** 2021-08-30

**Authors:** Cecilia Marini, Vanessa Cossu, Mandeep Kumar, Marco Milanese, Katia Cortese, Silvia Bruno, Grazia Bellese, Sonia Carta, Roberta Arianna Zerbo, Carola Torazza, Matteo Bauckneht, Consuelo Venturi, Stefano Raffa, Anna Maria Orengo, Maria Isabella Donegani, Silvia Chiola, Silvia Ravera, Patrizia Castellani, Silvia Morbelli, Gianmario Sambuceti, Giambattista Bonanno

**Affiliations:** 1 CNR Institute of Molecular Bioimaging and Physiology (IBFM), Segrate, 20054 Milan, Italy; 2 Nuclear Medicine, IRCCS Ospedale Policlinico San Martino, 16132 Genoa, Italy; matteo.bauckneht@hsanmartino.it (M.B.); annamaria.orengo@hsanmartino.it (A.M.O.); silvia.chiola@gmail.com (S.C.); silviadaniela.morbelli@hsanmartino.it (S.M.); sambuceti@unige.it (G.S.); 3 Department of Health Sciences, University of Genoa, 16132 Genoa, Italy; vane.6291@gmail.com (V.C.); Stefanoraffa@live.com (S.R.); isabella.donegani@gmail.com (M.I.D.); 4 Department of Pharmacy, Section of Pharmacology and Toxicology, University of Genoa, 16148 Genoa, Italy; mandeep.pharm@gmail.com (M.K.); marco.milanese@unige.it (M.M.); 3921690@studenti.unige.it (R.A.Z.); torazza@difar.unige.it (C.T.); bonanno@difar.unige.it (G.B.); 5 Department of Experimental Medicine, Human Anatomy, University of Genoa, 16132 Genoa, Italy; cortesek@unige.it (K.C.); silvia.bruno@unige.it (S.B.); bgrazia@unige.it (G.B.); consueloventuri68@gmail.com (C.V.); silvia.ravera@unige.it (S.R.); 6 Cell Biology Unit, IRCCS Ospedale Policlinico San Martino, 16132 Genoa, Italy; sonia.carta@hsanmartino.it (S.C.); patrizia.castellani@hsanmartino.it (P.C.); 7 Pharmacology and Toxycology, IRCCS Ospedale Policlinico San Martino, 16132 Genoa, Italy

**Keywords:** Amyotrophic Lateral Sclerosis (ALS), SOD1^G93A^, astrocytes, endoplasmic reticulum, redox stress, H6PD, pentose phosphate pathway, FDG-PET/CT, spinal cord, motor cortex

## Abstract

Recent studies reported that the uptake of [18F]-fluorodeoxyglucose (FDG) is increased in the spinal cord (SC) and decreased in the motor cortex (MC) of patients with ALS, suggesting that the disease might differently affect the two nervous districts with different time sequence or with different mechanisms. Here we show that MC and SC astrocytes harvested from newborn B6SJL-Tg (SOD1^G93A^) 1Gur mice could play different roles in the pathogenesis of the disease. Spectrophotometric and cytofluorimetric analyses showed an increase in redox stress, a decrease in antioxidant capacity and a relative mitochondria respiratory uncoupling in MC SOD1^G93A^ astrocytes. By contrast, SC mutated cells showed a higher endurance against oxidative damage, through the increase in antioxidant defense, and a preserved respiratory function. FDG uptake reproduced the metabolic response observed in ALS patients: SOD1^G93A^ mutation caused a selective enhancement in tracer retention only in mutated SC astrocytes, matching the activity of the reticular pentose phosphate pathway and, thus, of hexose-6P dehydrogenase. Finally, both MC and SC mutated astrocytes were characterized by an impressive ultrastructural enlargement of the endoplasmic reticulum (ER) and impairment in ER–mitochondria networking, more evident in mutated MC than in SC cells. Thus, SOD1^G93A^ mutation differently impaired MC and SC astrocyte biology in a very early stage of life.

## 1. Introduction

Recent evidence indicates that the degeneration of upper and lower motor neurons, as exhibited in amyotrophic lateral sclerosis (ALS), represents a non-cell-autonomous disease at least partially modulated by astrocytes [1,2]. Indeed, the enhanced content of reactive oxygen species (ROS) [3,4] in these glial cells exceeds the neuronal damage in ALS patients and precedes the motor impairment in experimental disease models [5,6,7,8].

The absence of methods to noninvasively evaluate redox stress in the central nervous system did not permit the estimation of the role of astrocyte dysfunction in the progression of ALS in patients. Nevertheless, recent evidence indicates a direct relationship between [18F]-fluorodeoxyglucose (FDG) uptake and oxidative stress, reporting a direct and strict link between tracer retention and the activation of a specific pentose phosphate pathway (PPP) dedicated to the reduction of NADP to NADPH [9]. This pathway is triggered by the “omnivore” enzyme hexose-6P dehydrogenase (H6PD) [10,11,12], which can process FDG and FDG6P, as well as many phosphorylated and free hexoses within the endoplasmic reticulum (ER). Indeed, ER-PPP has been found to account for FDG uptake in cancer cells [12,13,14], skeletal muscles [15,16], cardiomyocytes [17] and, more importantly, neurons and astrocytes [18]. Accordingly, the predictive power of high FDG uptake in the spinal cord (SC), recently documented in symptomatic ALS patients [19], might reflect the capability of PET/CT to point out the enhanced redox stress in the environment surrounding the lower motor neurons. Interestingly, this same observation faced a decreased tracer retention in the motor cortex (MC) [20], suggesting that the divergent FDG uptake in MC and SC might indicate the presence of different metabolic patterns in the environments of upper and lower motor neurons. However, its observation in symptomatic patients might also reflect a different time course of the damage featuring ALS progression. At this time, the relatively low MC tracer retention might indeed reflect the consequence of cortical atrophy and the loss of metabolically active cells because of a long-standing exposure to the disease-related oxidative injury as opposed to a delayed appearance of redox damage in SC.

Thus, to assess the mechanisms underlying the different metabolic patterns of the MC and SC, the present study evaluated the redox balance, mitochondrial function, ultrastructure and FDG uptake of astrocytes harvested from the MC and SC of newborn SOD1^G93A^ mice.

## 2. Materials and Methods

### 2.1. Animals

B6SJL-Tg(SOD1*G93A)1Gur mice expressing high copy number of mutant human SOD1 with a Gly93Ala substitution (SOD1^G93A^ mice) [21] were originally obtained from Jackson Laboratories (Bar Harbor, ME, USA) and maintained by crossing *SOD1^G93A^* male mice with background-matched B6SJL wildtype (WT) females. SOD1^G93A^ mice represent the most widely used animal model for ALS preclinical studies [22] since it recapitulates several pathological hallmarks of ALS in human patients. Mice carrying the SOD1^G93A^ mutation were identified by analyzing the tissue extracts from tail tips [23]. Briefly, tissue was homogenized and freeze–thawed twice. SOD1 was evaluated by staining for its enzymatic activity on 10% non-denaturing polyacrylamide gel electrophoresis by incubation for 15 min under shaking with 4-nitro blue tetrazolium chloride (NTB; Merck, Darmstadt, Germany) and then with riboflavin (Merck, Darmstadt, Germany), supplemented with tetramethylethylenediamine (Carl Roth GmbH Karlsruhe, Germany). Gels were illuminated with a white-light box for 10–15 min: under light exposure, riboflavin is reduced, leading to the production of O_2_^−^, which reduces NBT to form formazan, a dark blue color precipitate.

Experiments were conducted in accordance with the European Communities Council Directive (EU Directive 114 2010/63/EU for animal experiments) and with the Italian D.L. No. 26/2014 and were approved by the local ethical committee and by the Italian Ministry of Health (project authorization No. 97/2017-PR). All efforts were made in minimizing animal suffering and using the minimal number of animals necessary to produce statistically reliable results.

### 2.2. Astrocyte Preparation

Astrocytes were isolated from the MC and SC of 2-day-old SOD1^G93A^ or WT mice according to Paluzzi and colleagues with some modifications [24]. Two days after birth, pups (P1-2) were anesthetized and sacrificed by cervical dislocation to remove MC and SC under binocular dissection (Nikon SMZ-2T, Japan,) in HBSS at 4 °C. Each dissected SC was gently homogenized in 1 mL Dulbecco’s modified Eagle medium (DMEM; Euroclone, Cat# ECM0728L) containing 10% fetal bovine serum (Euroclone, Cat# ECS0180L), 1% glutamine (Euroclone, Cat# ECB3004D) and 1% penicillin/streptomycin (Euroclone, Cat# ECB3001D). Then, tissue suspension was seeded in a 35 mm Petri dish (Euroclone, Cat# ET2035), precoated with poly-L-ornithine hydrochloride (1.5 µg/mL; Sigma, Cat# P2533) and laminin (3 µg/mL; Sigma, Cat# L2020). MC was isolated from the brain under binocular dissection (Nikon SMZ-2T, Japan, CAT# 2049) and homogenized in 8 mL of complete DMEM as above. Tissue suspension was seeded in two 25 cm^2^ flasks (Euroclone, Cat# ET7026). Samples were placed at 37 °C in humidified 5% CO2 incubator, and the medium containing tissue fragments was replaced with fresh complete DMEM after 24 h and then every 48 h. After 7 days in vitro (DIV), astrocytes were shaken for 15 min to remove microglia cells, detached by Trypsin-EDTA (Euroclone, Cat# ECB3052B), replated and cultured to confluence. After 15 DIV, cell cultures were again shaken for 15 min, and astrocytes were collected for the experiments. For immunofluorescence (IF) studies, astrocytes were detached after 7 DIV, as described above, and replated onto 12 mm diameter poly-L-ornithine and laminin precoated glass coverslips placed at the bottom of 24-well plates. After 15 DIV, astrocytes were processes for IF staining.

### 2.3. Flow Cytometry

MC and SC neonatal astrocytes from WT or SOD1^G93A^ pups were detached by trypsin-EDTA and centrifuged for 5 min at 500× *g*. About 5 × 10^5^ cells were resuspended and saturated for unspecific bonds by incubating with 0.5% bovine serum albumin (BSA) in phosphate-buffered saline (PBS, pH 7.4) for 15 min at RT. Aliquots of the suspension were stained (1 h at RT) with the following fluorochrome-conjugated antibodies for flow cytometry: mouse monoclonal anti-GFAP antibody conjugated with Alexa Fluor A488 (Thermo Fisher Scientific, Cat# 53-9892-82), rat monoclonal anti-ACSA2 antibody conjugated with phycoerythrin (PE) (Miltenyi Biotec, Cat# 130-102-365) and rat monoclonal anti-TMEM119 antibody conjugated with Alexa Fluor A488 (Abcam, Cat# ab225497). For GFAP staining, cell suspensions were previously fixed and permeabilized, 20 min at 4 °C, by using the Cytofix/Cytoperm Fixation/Permeabilization Kit (BD Bioscience, Cat# 554714). After staining, cells were centrifuged (5 min at 500× *g*) and pellets were resuspended in PBS for flow cytometry analyses. Cell debris and dead cells were excluded from analysis by 7-aminoactinomycin D (7-AAD) labeling. Data were acquired on a Guava easyCyte 6 flow cytometer (Merck Millipore, Burlington, MA, USA) and processed using the GuavaSoft 3.1.1 software (Merck Millipore).

### 2.4. Confocal Microscopy Immunofluorescence

SC and MC neonatal astrocytes from WT or SOD1^G93A^ pups were cultured onto 12 mm glass coverslips. Cells were washed three times with PBS and postfixed with 4% paraformaldehyde (Sigma-Aldrich, Cat# 47608) in PBS for 15 min at RT. Cells were permeabilized with methanol for 5 min at −20 °C, washed three times with PBS and saturated with 0.5% BSA in PBS for 15 min at RT. Samples were incubated with mouse monoclonal anti-glial fibrillary acid protein (GFAP, 1:500; Sigma Aldrich, Cat# G3893) and rat monoclonal anti-integrin alpha-M/beta-2 (CD11b, 1:500; Abcam, Cat# ab8878) primary antibodies overnight at 4 °C. Astrocytes were then washed three times with 0.5% BSA in PBS and incubated with donkey anti-mouse Alexa Fluor A488-conjugated (1:3000; Thermo Fisher Scientific, Cat# R37118) and goat anti-rat Alexa Fluor A647-conjugated (1:3000; Thermo Fisher Scientific, Cat# A-21247) secondary antibodies for 1 h at RT. Cells were washed three times with PBS, and coverslips were assembled on microscopy glass slides by using ProLong Gold antifade mountant (Thermo Fisher Scientific, Cat# P10144). Fluorescence image (512 × 512 × 8 bit) acquisition was performed by a three-channel Leica TCS SP5 laser-scanning confocal microscope, equipped with 458, 476, 488, 514, 543 and 633 nm excitation lines, through a plan apochromatic oil immersion objective 63x (1.4 NA). Light collection configuration was optimized according to the combination of chosen fluorochromes, and sequential channel acquisition was performed to avoid crosstalk. Leica “LAS AF” software package was used for image acquisition.

### 2.5. Enzymatic Assays

Cultured cells were centrifuged at 1000× *g* for 2 min to remove the growth medium. The pellet was suspended in PBS supplemented by protease inhibitors. Obtained homogenates were thus sonicated twice for 10 s in ice, with a break of 30 s. The activity of H6PD and glucose-6-phosphate dehydrogenase (G6PD) was assayed using a double-beam spectrophotometer (UNICAM UV2, Analytical S.n.c., Italy) to follow the reduction of NADP at 340 nm [11,12,13]. H6PD enzymatic function was tested in the presence of Tris-HCl pH 7.4 100 mM, 2-deoxyglucose-6P (2DG6P) 10 mM and NADP 0.5 mM. By contrast, G6PD activity was assayed in the presence of Tris-HCl pH 7.4 100 mM, G6P 10 mM and NADP 0.5 mM. Complex I activity was assayed following the reduction of ferrocyanide (FeCN), at 420 nm, using the following solution: TRIS7.4, NADH 0.6 mM, antimycin 50 µM and FeCN 0.8 mM. Antioxidant capacity was evaluated following the instructions of the manufacturer of a dedicated kit (Abcam; Cat #ab65329) that provides a complete description of the total cell antioxidant power associated with the endogenous scavengers, expressed as Trolox equivalent antioxidant capacity content [14,16]. Similarly, NADP/NADPH ratio was tested using a dedicated Assay Kit (Abcam; Cat#ab65349), following the manufacturer’s instructions. Finally, malondialdehyde (MDA) levels were evaluated by the thiobarbituric acid reactive substances assay [16,25]. In all cases, enzymatic activity was normalized for total protein concentrations tested using Bradford analysis [26].

### 2.6. Seahorse Analysis

Astrocyte oxygen consumption rate (OCR) and extracellular acidification rate (ECAR) were determined using a Seahorse XFp Extracellular Flux Analyzer (Agilent Technologies). Twenty-four hours prior to the assay, 10,000 astrocytes/well were seeded in XF plates. OCR and ECAR were monitored according to the manufacturer’s instructions. Briefly, the day of the experiment, the medium was replaced with Agilent Seahorse DMEM, pH 7.4, enriched with glucose (11 mM), glutamine (2 mM) and pyruvate (1 mM). Three measurements of OCR and ECAR were taken for the baseline and after sequential injection of the ATP-synthase inhibitor oligomycin A (1 µM) and the ATP synthesis uncoupler carbonyl cyanide-4-trifluoromethoxyphenylhydrazone (FCCP) (0.8 µM).

### 2.7. Cytofluorimetric Analysis

Both MC and SC astrocytes (3 × 10^7^ cells) from wildtype or SOD1G93A mice were stained with 5 μM 2′,7′-dichloro-dihydrofluorescein diacetate (H_2_DCFDA), 1 μM MitoTracker Red and 5 μM MitoSOX Red (all from Invitrogen by Thermo Fisher Scientific, Waltham, MA, USA). The astrocytes were then washed two times with PBS, centrifuged for 5 min at 1200 rpm and resuspended in PBS + 1% BSA for cytofluorimetric analysis. All experiments were performed in triplicate, and analysis was done on a FACScan (Becton Dickinson, Milan, Italy).

### 2.8. In Vitro Extraction Fraction of FDG

In vitro FDG uptake of astrocytes was evaluated using the LigandTracer White instrument (Ridgeview, Uppsala, SE) according to our previously validated procedure [13,17,27]. Briefly, the device consists of a beta-emission detector and a rotating platform harboring a standard Petri dish. The rotation axis is inclined at 30° from the vertical so that the organ alternates its position from the nadir (for incubation) to the zenith (for counting) every minute. For each group, 600,000 astrocytes were seeded the day before the experiments under standard conditions. Soon before the experiment, culture medium was replaced with DMEM containing glucose at 5.5 mM and enriched with 1.8 to 2.2 MBq/mL FDG. Tracer kinetic uptake was measured over 120 min of experiments. All experiments were performed in triplicate, and data were normalized for cell number.

### 2.9. Western Blot Analysis

Astrocyte whole-cell lysates were prepared using EB Lysis Buffer (HEPES pH 7.4 20 mM, NaCl 150 mM, glycerol 10% and Triton X-100 1%) with protease inhibitor cocktail and phosphatase inhibitors (PhosStop) (Roche, Basel, Switzerland) and sodium orthovanadate. Petri dishes were scraped to collect the whole lysates and incubated on ice for 15 min. Lysates were finally centrifuged for 5 min at 13,200 rpm at 4 °C to remove cellular debris. Protein cell extracts and SDS polyacrylamide gel electrophoresis (NW04120box, BOLT Bis-Tris plus 4–12%, Invitrogen) were performed using standard protocols. Proteins were detected with ECL Detection Reagent (BioRad, Hercules, CA, USA). Protein quantification was performed using Bradford protein assay (BioRad, Hercules, CA, USA). Anti-vinculin (Sigma, V9131) was used as loading control. We tested the following antibodies: anti-cytochrome c (Santa Cruz Biotechnology, inc., H-104, Cat# sc-7159) and anti-mitofusin 2 (Mfn2, Thermo Fisher, Cat# PA5-72811).

Secondary antibodies were horseradish peroxidase-conjugated anti-mouse (Cat# G21040) and anti-rabbit (Cat# G21234) (Molecular Probes, Thermo Fisher Scientific, Waltham, MA, USA), and the detection of proteins was performed with ECL Detection Reagent (BioRad, Hercules, CA, USA).

### 2.10. Ultrastructural Analysis

For transmission electron microscopy (TEM), astrocytes were fixed in 0.1 M cacodylate buffer containing 2.5% glutaraldehyde (Electron Microscopy Science, Hatfield, PA, USA), for 2 h at room temperature. Samples were postfixed in osmium tetroxide for 1 h and 1% uranyl acetate for 2 h. Samples were next dehydrated through a graded ethanol series and embedded in epoxy resin (Poly-Bed; Polysciences, Inc., Warrington, PA, USA) overnight at 42 °C and for 2 days at 60 °C. Ultrathin sections (50 nm) were then counterstained with 5% uranyl acetate and lead citrate and observed with an HT7880 device (Hitachi, Japan). Digital images were captured with a MegaView 3 camera. Analysis of the size of morphologically identified mitochondria and rough ER was performed with EMSIS software package (EMSIS, Germany), and data were plotted as histograms.

Morphometric measurements were carried out using ImageJ (National Institutes of Health). ER–mitochondria distance was analyzed only when it was <50 nm [28]. Using this cut-off, *n* > 5 mitochondria per image in 70 images per condition were considered. Visual inspection of TEM images was performed by investigators blinded to sample identity. ER–mitochondrion tethers were identified by an interorganelle distance of ≤30 nm [29]. In MC astrocytes, the ER–mitochondrion relationship could be estimated in 46 and 45 points for WT and SOD1^G93A^ samples. For SC, these numbers were 48 and 40, respectively.

### 2.11. Statistical Analysis

All experimental groups were studied in triplicate. Data are presented as mean ± standard deviation (SD). Differences among the experimental conditions were tested using one-way analysis of variance (ANOVA), and the Tukey test was used to test the statistical hypothesis. Significance was considered for *p* values <0.05. All analyses were performed using SPSS software Advanced Models 15.0 (Chicago, IL, USA).

## 3. Results

### 3.1. Purity of WT and SOD1^G93A^ Newborn Astrocyte Cell Cultures

**Figure 1:**
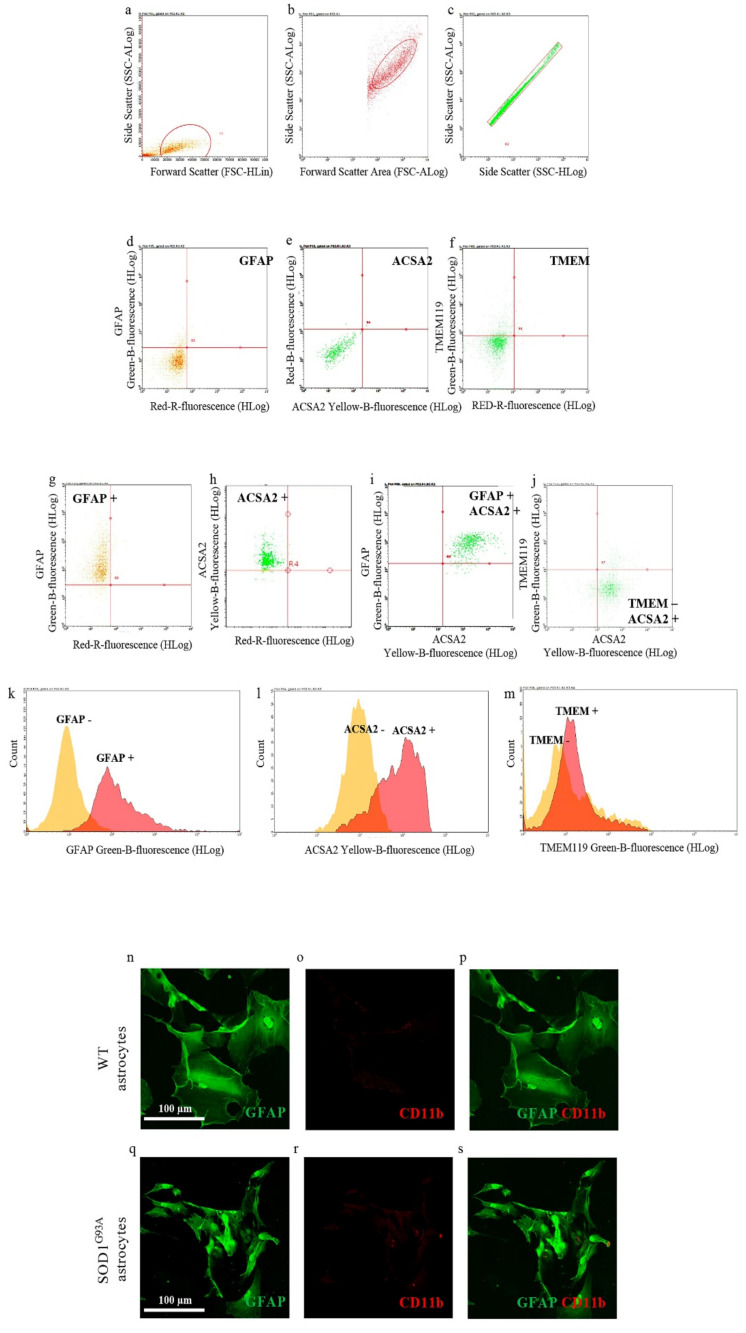
Astrocyte cell culture purity. Spinal cord neonatal astrocyte primary cell cultures were prepared from WT and SOD1^G93A^ P2 pups, and their purity has been verified by flow cytometry (**a**–**m**) and immunofluorescence (**n**–**s**). (**a**–**c**) Representative flow cytometry dot plots of SOD1^G93A^ astrocytes showing the cell population gated by (**a**) side scatter (SSC-ALog) vs. forward scatter (FSC-HLin), (**b**) SSC-ALog vs. forward scatter (FSC-ALog) and (**c**) SSC-Alog vs. side scatter (SSC-HLog). (**d**–**f**) Representative dot plots of SOD1^G93A^ unstained SOD1^G93A^ astrocytes. (**g**–**j**) Representative dot plots of SOD1^G93A^ astrocytes after incubation with fluorophore-conjugated antibodies for (**g**) GFAP (astrocyte marker; green fluorescence), (**h**,**i**) ACSA2 (astrocyte marker; yellow fluorescence) and (**j**) TMEM119 (microglia marker; green fluorescence). (**k**–**m**) Representative flow cytometry histogram plots of SOD1^G93A^ astrocytes showing the expression of (**k**) GFAP, (**l**) ACSA2 and (**m**) TMEM, compared to the respective unstained controls. For quantization, *n* = 4 biological replicates per group were analyzed. (**m**–**r**) Representative confocal microscopy images of WT and SOD1^G93A^ spinal cord neonatal astrocyte primary cell cultures stained with selective antibodies for (**n**,**q**) GFAP (green fluorescence) and (**o**,**r**) CD11b (specific microglia marker; red fluorescence); (**p**,**s**) merge panels. For quantization, *n* = 3 biological replicates per group were analyzed, with each value defined in triplicate.

In the first set of experiments, we investigated the purity of neonatal astrocytes cultured from the spinal cord of SOD1^G93A^ P2 mouse pups by flow cytometry, labeling cell suspension with antibodies for GFAP or ACSA2, specific astrocyte markers [30], and TMEM119, a specific microglia marker [31]. Figure 1g–m shows that astrocyte preparations indeed express both the astroglial markers GFAP and ACSA2 (95.03 ± 3.38% GFAP-positive cells, Figure 1g,k; 94.46 ± 1.92% ACSA2-positive cells, Figure 1h,l; 98.34 ± 1.42% GFAP and ACSA2 coexpressing positive cells, Figure 1i) when compared to the respective unstained controls (Figure 1d,e). Moreover, cell suspensions showed very low contamination of microglia cells, labeled with TMEM119 (2.42 ± 0.69% TMEM119-positive cells, Figure 1j,m), compared to the respective unstained control (Figure 1f).

We also performed confocal microscopy studies staining WT and SOD1^G93A^ spinal cord neonatal astrocytes with antibodies for GFAP and integrin alpha-M/beta-2 (CD11b; specific microglia marker). Representative images reported in Figure 1n–s indicate that both WT and SOD1^G93A^ astrocytes are efficiently stained with GFAP (green fluorescence), while they do not show contamination by microglia cells, labeled with CD11b (red fluorescence).

Overall, these results indicate that the neonatal astrocyte primary cell culture preparations used here were not contaminated by microglia cells.

### 3.2. Redox Stress and Antioxidant Response in Cortical and Spinal SOD1^G93A^ Newborn Astrocytes

**Figure 2:**
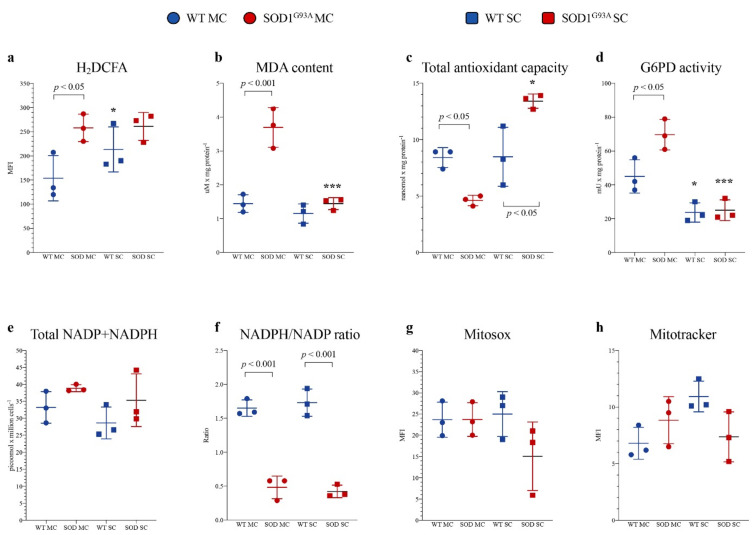
Astrocyte antioxidant response and oxidative stress. (**a**) ROS levels measured using H_2_DCFDA mean fluorescence index (MFI), (**b**) malondialdehyde content (MDA), (**c**) antioxidant capacity and (**d**) G6PD activity in wildtype (blue) and SOD1^G93A^ (red) astrocytes harvested from motor cortex (MC) and spinal cord (SC). (**e**) NADPH level in wildtype (light blue) and SOD1^G93A^ (pink) astrocytes and NADP^+^ level in wildtype (blue) and mutated cells (red). (**f**) NADPH/NADP^+^ ratio in wildtype (blue) and SOD1^G93A^ (red) astrocytes. (**g**) Mean fluorescence intensity (MFI) of MitoSOX and (**h**) MitoTracker in wildtype (blue) and SOD1^G93A^ (red) astrocytes harvested from MC and SC. Data are shown as the mean ± SD. *n* = 3 experiments per group, with each value defined in triplicate. * = *p* < 0.05, *** = *p* < 0.01 (WT SC astrocytes vs. WT MC astrocytes and SOD1^G93A^ SC astrocytes vs. SOD1^G93A^ MC astrocytes).

Astrocytes were isolated from the MC and SC of SOD1^G93A^ mice and WT littermates. In SOD1^G93A^ MC astrocytes, the intensity of H_2_DCFDA fluorescence was higher with respect to the corresponding WT, indicating a selective increase in ROS content (Figure 2a), as confirmed by a significant increase in lipid peroxidation evaluated by MDA levels (Figure 2b). The enhanced redox stress at least partially reflected an inadequate response of antioxidant pathways. Indeed, total antioxidant capacity was decreased in MC SOD1^G93A^ astrocytes with respect to their WT counterpart (Figure 2c). In eukaryotic cells, ROS scavenging largely involves the availability of NADPH reductive power whose main source is represented by the cytosolic PPP [32]. The catalytic function of its triggering enzyme G6PD, evaluated by spectrophotometric assay, was actually increased in mutated MC astrocytes (*p* < 0.05, Figure 2d). Then, obtained data suggest an acceleration of cytosolic PPP that should be associated with an enhanced reduction of NADP to NADPH. Nevertheless, the overall NADPH content and the NADPH/NADP ratio were remarkably decreased in MC astrocytes (Figure 2e,f), suggesting that the high-rate utilization of this reducing cofactor was not counterbalanced by cytosolic PPP, despite the enhanced activity of its triggering enzyme.

Redox balance was remarkably different in SC cultures. Indeed, H_2_DCFDA staining intensity and thus ROS content were higher in SC WT astrocytes with respect to MC WT ones (Figure 2a). This difference was, however, not paralleled by an increased lipid peroxidation, suggesting a higher endurance against the redox stress in the spinal district of WT mice (Figure 2b). This pattern was virtually abolished in the experimental ALS model. Indeed, H_2_DCFDA fluorescence of SC SOD1^G93A^ astrocytes was markedly increased and became similar to the intensity observed in corresponding MC cultures. Nevertheless, MDA content was only slightly increased (Figure 2a,b). These findings nicely agreed with the behavior of total antioxidant capacity that was selectively enhanced in mutated SC astrocytes (Figure 2c).

This response was at least partially independent of the cytosolic PPP since G6PD activity of WT SC cells was significantly lower than in MC cells and was virtually not affected by SOD1^G93A^ mutation (Figure 2d). A different behavior was displayed by H6PD: its activity was virtually undetectable in all cultures except in mutated SC astrocytes in which its activity accounted for 4.97 ± 0.8 mU/mg of proteins. However, the NADPH content and the NADPH/NADP ratio were similarly decreased with respect to mutated MC astrocytes (Figure 2e,f), suggesting that the high-rate utilization of this reducing cofactor was not counterbalanced by cytosolic or reticular PPP activity (Figure 2d–f).

It is well known that mitochondria play a double role in oxidative stress, representing, at the same time, a main source of ROS and a primary target of ROS-induced damage. We thus estimated the mitochondrial redox status, using MitoSOX Red, a specific and mitochondrial-targeted detection probe for superoxide radical (O_2_^−^). As reported in Figure 2g, fluorescence intensity was comparable in mutated vs. WT MC or SC cultures. Similarly, the affinity for the mitochondrial probe was only slightly, and not significantly, decreased by SOD1^G93A^ mutation in SC astrocytes with respect to both mutated MC and WT SC cells (Figure 2g).

A similar conclusion also applied to the evaluation of mitochondrial membrane polarization. Indeed, MitoTracker Red staining provided largely variable results without any significant difference among all groups of tested cultures (Figure 2h).

Altogether, these data thus indicated that SOD1^G93A^ mutation is associated with an early enhancement of redox stress that selectively affects MC astrocytes harvested 2 days after birth. However, this oxidative environment seems to not be explained by mitochondrial damage, indicating the possible role of other cell structures or functions in the redox impairment of MC astrocytes.

### 3.3. Oxidative Phosphorylation Coupling and Glycolytic Flux in Cortical and Spinal SOD1^G93A^ Newborn Astrocytes

**Figure 3:**
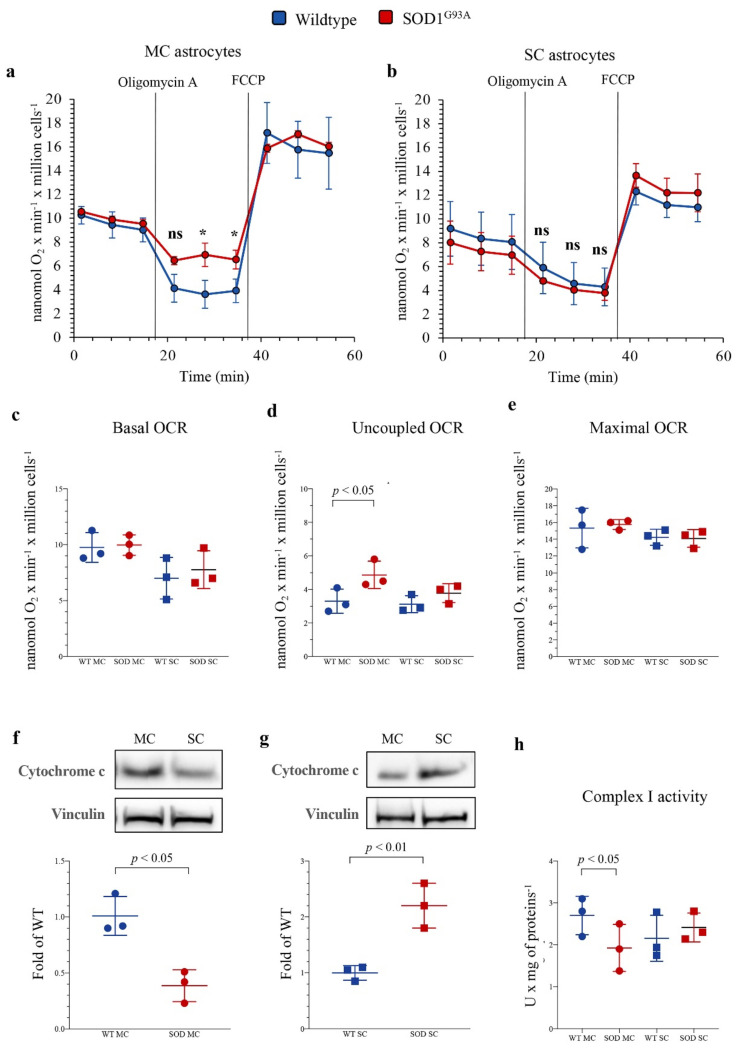
*Mitochondrial energetic function in SOD1^G93A^ astrocytes.* Basal oxygen consumption rate (OCR) measured in presence of glucose (11 mM), glutamine (2 mM) and pyruvate (1 mM) and after sequential injection of the ATP-synthase inhibitor oligomycin A (1 mM) and the ATP synthesis uncoupler FCCP (0.8 µM) in (**a**) motor cortex (MC) and (**b**) spinal cord (SC) astrocytes, in wildtype (blue) and SOD1^G93A^ astrocytes (red). (**c**) Basal, (**d**) uncoupled and (**e**) maximal OCRs in wildtype (blue) and SOD1^G93A^ (red) astrocytes harvested from MC and SC. Western blot analysis and relative densitometric quantitative analyses of cytochrome c in MC (**f**) and SC (**g**) astrocytes. (**h**) Complex I activity in wildtype (blue) and SOD1^G93A^ (red) astrocytes harvested from MC and SC. Data are shown as the mean ± SD. *n* = 3 experiments per group, with each value defined in triplicate. ns = not significant, * = *p* < 0.05 vs. corresponding WT astocytes.

To evaluate the mechanisms underlying the apparent mismatch of increased redox damage not paralleled by evident mitochondrial alterations, we extended our evaluation to the respiratory function and glycolytic flux, using the Seahorse technology. In newborn MC astrocytes, SOD1^G93A^ mutation left unaltered both baseline and maximal oxidative phosphorylation (OXPHOS) rates evaluated under control conditions and during respiratory uncoupling by FCCP (Figure 3a,c,e). However, the mutation caused a relative respiratory uncoupling since OCR under ATP-synthase blockade by oligomycin was higher in mutated than in WT MC cultures (Figure 3a,d), suggesting a relative decrease in OXPHOS-linked ATP production. This abnormality was markedly less evident in SC astrocytes, in which SOD1^G93A^ mutation left unaltered basal, ATP-linked and maximal OCRs (Figure 3b–e).

**Figure 4:**
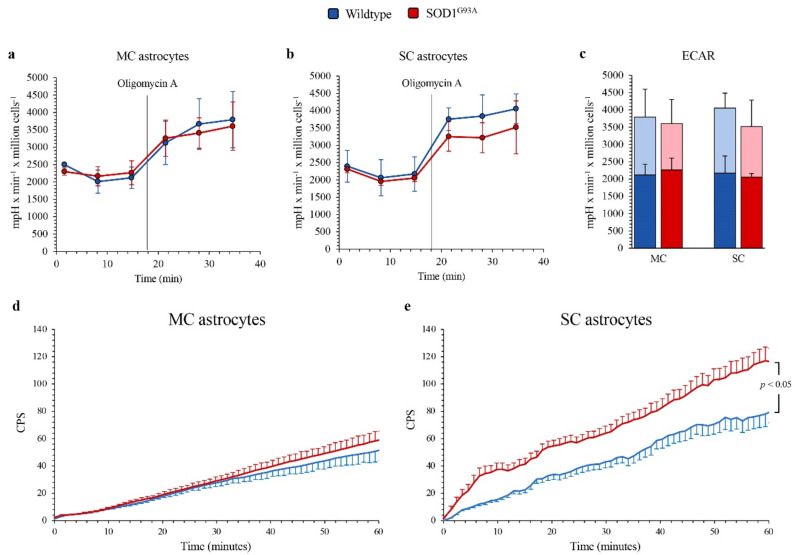
*Glycolytic flux and FDG uptake in SOD1^G93A^ astrocytes.* Basal extracellular acidification rate (ECAR) measured in presence of glucose (11 mM), glutamine (2 mM) and pyruvate (1 mM) and after injection of the ATP-synthase inhibitor oligomycin A (1 mM) in (**a**) motor cortex (MC) and (**b**) spinal cord (SC) astrocytes, in wildtype (blue) and SOD1^G93A^ astrocytes (red). (**c**) Basal (blue and red in wildtype and SOD1^G93A^ astrocytes, respectively) and maximal ECARs (light blue and pink in wildtype and SOD1^G93A^ astrocytes, respectively) in MC and SC cells. Average time-course of in vitro radioactivity expressed as a count per second (CPS) of the FDG dose measured by the LigandTracer White device, in MC (**d**) and SC (**e**) astrocytes. Data are shown as the mean ± SD. *n* = 3 experiments per group, with each value defined in triplicate.

The differential OXPHOS efficiency of the two central nervous system regions was reproduced by the Western blot analysis of electron transport between complexes III and IV. Indeed, SOD1^G93A^ genotype was associated with a significant decrease in total cytochrome c levels in MC astrocytes, as opposed to an increase in SC ones (Figure 3f,g). In addition, complex I activity function was significantly decreased in SOD1^G93A^ MC astrocytes, while it remained virtually unchanged in SC cultures (Figure 3h). In agreement with the relatively small contribution of mitochondria to astrocyte energy metabolism, the selective OXPHOS impairment observed in MC mutated astrocytes was not associated with any response of glycolytic flux. Indeed, extracellular ECAR was remarkably similar in all cultures, irrespective of source type (mutated or WT), district (MC or SC) or condition (baseline or respiratory inhibition by oligomycin) (Figure 4a–c).

### 3.4. Mismatch between FDG Uptake and Glycolytic Flux

The absent effect of SOD1^G93A^ mutation on glycolytic flux, estimated by ECAR, was not paralleled by the response of FDG kinetic uptake, evaluated by the LigandTracer method. Indeed, it was remarkably similar in all cell cultures except in mutated SC astrocytes in which tracer retention was significantly enhanced (Figure 4d,e).

This selective response of tracer retention was obviously independent of G6PD catalytic function that, as previously described, was higher and further increased by SOD1^G93A^ mutation only in MC astrocytes. By contrast, the enhanced FDG uptake at least partially matched the increased activity of H6PD, whose catalytic function was assayable, and thus over the method threshold, only in SOD1^G93A^ SC cultures.

These data thus extended to the astrocytes cultured from these experimental ALS models the previous evidence about the link between FDG uptake and ER-PPP activation in the local response to redox stress [16,17].

### 3.5. SOD1^G93A^ Mutation and ER–Mitochondria Network in MC and SC Astrocytes

**Figure 5:**
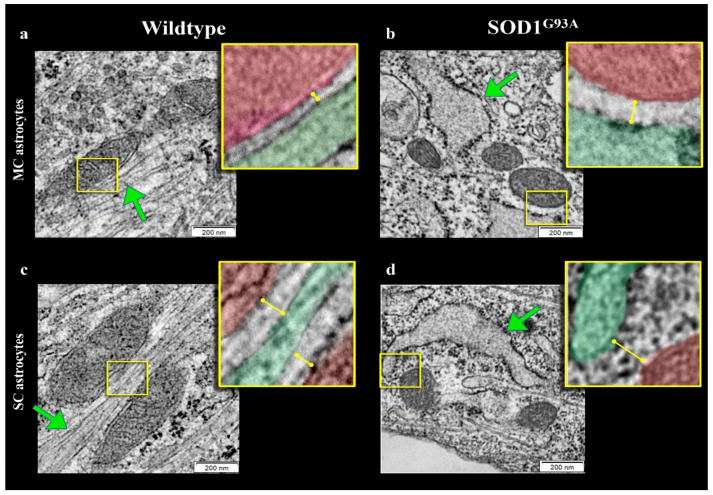
*Electron microscopy images of ER–mitochondria networking.* Representative electron microscopy images of motor cortex (MC) astrocytes harvested from wildtype (**a**) and SOD1^G93A^ mice (**b**) and astrocytes isolated from spinal cord (SC) of wildtype (**c**) and mutated (**d**) mice. Mitochondria and ER profiles are marked in red and green, respectively.

**Figure 6:**
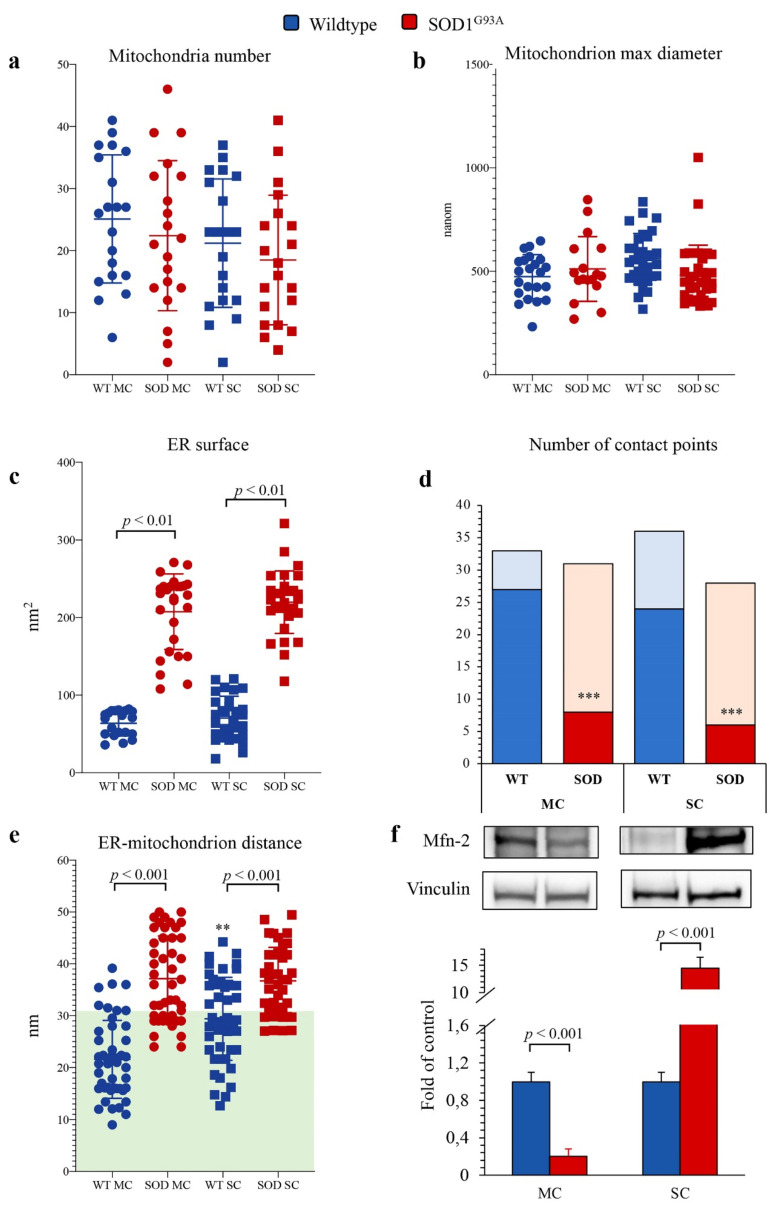
*Electron microscopy ultrastructure of ER and mitochondria.* Mitochondrial number (**a**), mitochondria max diameter (**b**), ER surface (**c**) in wildtype (blue) and SOD1^G93A^ (red) of motor cortex (MC, circles) and spinal cord (SC, squares) astrocytes. Number of contact points (**d**) and distance between ER and mitochondria (**e**) evaluated in MC and SC astrocytes harvested from WT and SOD1^G93A^ mice (with the green area indicating distance < 30 nm, considered as contact points). Western blot analysis and relative densitometry quantitative analyses of Mfn2 (**f**) in wildtype (blue) and SOD1^G93A^ (red) astrocytes harvested from MC and SC. Data are shown as the mean ± SD. *n* = 3 experiments per group, with each value defined in triplicate. ** = *p* < 0.01, *** = *p* < 0.001 vs. corresponding MC astrocytes.

Several studies documented that mitochondria and ER network is necessary for the preservation of cellular homeostasis under stress conditions [33,34,35,36]. We thus evaluated the possible presence of abnormalities in mitochondrial number, size and connections within the ER in astrocytes harvested from newborn SOD1^G93A^ mice. TEM showed that mitochondria morphology was normal with well-defined cristae and membrane structure in both MC and SC astrocytes regardless of the genotype (Figure 5a–d and Figure 6a,b).

By contrast, SOD1^G93A^ mutation was associated with an impressive enlargement of the ER in both MC and SC astrocytes (Figure 5b,d and Figure 6c). This alteration was associated with an impairment in ER–mitochondria networking. Indeed, the number of connections between these two organelles (defined as an intermembrane distance <50 nm) was decreased in transgenic cultures irrespective of their source (MC or SC) (Figure 6d).

When these contact points were measured in WT cultures, the ER–mitochondrion space was significantly larger in SC than in MC astrocytes (Figure 5a,c and Figure 6e). SOD1^G93A^ mutation virtually abolished this gradient and increased intermembrane distance to a higher degree in MC than in SC cells (Figure 5b,d and Figure 6e).

ER–mitochondrion contact point is the site for the exchange of signals and substrates for these two organelles and thus represents the preferential site of mitochondria-associated membrane proteins (MAMs) whose function is at least partially modulated by the levels of Mfn2 [28]. SOD1^G93A^ mutation caused a differential response of Mfn2 amount that was decreased in MC astrocytes and markedly increased in SC ones (Figure 6f).

## 4. Discussion

The main finding of the present study is the divergent effect of SOD1^G93A^ mutation on the redox balance of MC and SC astrocytes harvested 2 days after birth and cultured for 10 days under standard conditions. This disparity was paralleled by the metabolic response since the boost in G6PD activity of MC mutated astrocytes faced the enhancement of H6PD catalytic function in corresponding SC ones. The ER-PPP activation empowered SOD1^G93A^ SC astrocytes with the capability to ameliorate the impairment of ER–mitochondria connection that was instead well evident in mutated MC cultures, despite a similar degree of ER enlargement.

In agreement with previous observations in different cells and tissues [12,13,14,15,16,17,18], the increase in H6PD catalytic function was indexed by an enhanced FDG uptake. Altogether, these observations indicate that the divergent tracer retention, observed in SC and MC of symptomatic ALS patients, at least partially reflects a selective response of ER metabolism to the redox stress associated with SOD1^G93A^ mutation. The link between ER-PPP activation and tolerance to irreversible oxidative stress in the environments surrounding the upper and lower motor neurons configures the reticular redox balance as a possible target to better understand the mechanisms underlying ALS progression.

### 4.1. FDG Uptake and Astrocyte Redox Damage in Newborn SOD1^G93A^ Mice

Since the seminal paper by Sokoloff et al. in 1977 [37], FDG uptake has been considered an index of overall glucose consumption because, after entering the cytosol through GLUT-facilitated transmembrane transport, this tracer is phosphorylated to FDG6P that cannot be recognized by downstream enzymes channeling glucose to glycolysis or cytosolic PPP [38] and thus accumulates as a function of overall glucose phosphorylation, i.e., of overall glucose-6P (G6P) usage. Nevertheless, a measurable FDG loss has been observed in virtually all studied tissues, indicating the hydrolyzation of FDG6P by G6P-phosphatase [37]. The confinement of this enzyme within the ER has two important implications. On one side, it implies a carrier (identified as the G6P transporter or G6PT) able to transfer the polar FDG6P across the reticular membrane [39]. On the other side, it implies the presence of an enzymatic function able to prevent the hydrolytic reaction catalyzed by G6P-phosphatase. As confirmed by a series of studies [10,12,13,14,15,16,17,18], H6PD fits these characteristics due to its capability to process FDG6P and its reticular location.

The present data agree with the role of the H6PD reticular PPP in FDG uptake. Indeed, the selective increase in tracer retention of mutated SC astrocytes was associated with an enhancement in H6PD activity and with an invariance of both directly measured glycolytic flux and cytosolic PPP. By contrast, the enhancement of G6PD only occurred in newborn mutated MC cultures and was not associated with any change in FDG uptake, in agreement with the notion that this phosphorylated hexose is not recognized and thus processed by this enzyme [38].

Obtained data thus agree with our previous observation of SC lighting, opposed to a relative switch-off of the MC, in symptomatic ALS patients [20]. More importantly, they also confirm that the divergent FDG uptake of MC and SC districts reflects a different ER involvement empowering the reaction to the redox stress, thus extending to astrocytes the previous observation in skeletal muscles of SOD1^G93A^ mice. Indeed, the high susceptibility of MC astrocytes to oxidative damage might eventually accelerate their degeneration. Accordingly, the decreased tracer retention observed in the brain cortex of symptomatic ALS patients seems to rather reflect the consequence of cortical atrophy featuring the symptomatic disease phase [20]. In other words, this consideration characterizes FDG retention as a combined index of the metabolic activation of ER in each investigated volume of central nervous tissue, multiplied for the number of cells entailed in it.

### 4.2. Redox Damage and ER–Mitochondrion Networking in SOD1^G93A^ Mutation

In the present study, H6PD activity was considered a surrogate of PPP flux within the reticular lumen. This concept was justified by the notion that isolated ER membranes contain the enzymatic asset to manage the full sequence of PPP reactions [40]. This activity has been found to be ubiquitously distributed in all mammalian tissues [9], although relatively less represented in brain astrocytes [18]. In agreement with these previous observations, H6PD catalytic function remained under the detectability threshold of our method in all cultures except the mutated SC ones. On the other hand, its response confirms the notion that ER is empowered with specific pathways able to overcome the membrane impermeability and the consequent inaccessibility of NADPH reductive power, allowing the regulation of luminal redox balance.

The relevance of this empowerment is confirmed by the protective role of ER activation in the SC district against the irreversible consequences of the redox damage induced by SOD1^G93A^ mutation. Homogenate of MC astrocytes displayed a decreased function of complex I coupled by lower levels of cytochrome c. However, these abnormalities were only associated with moderate OXPHOS impairment in intact cells. Finally, MitoSOX and MitoTracker staining indicated a scarce contribution of mitochondrial ROS generation. On the other side, SC astrocytes displayed higher ROS levels with respect to MC ones also in WT mice, thus reproducing the finding of a higher O_2_^●–^ generation in SC naïve motor neurons compared with MC ones [41]. This difference was paralleled by a higher intermembrane distance of ER–mitochondria contact points in WT cultures that, however, partially prevented the detaching induced by SOD1^G93A^ mutation. Altogether, these data thus suggest the presence of different mechanisms underlying the antioxidant response in SC and MC astrocytes. In other words, the highly oxidative environment in normal SC seems to tailor the metabolic phenotype to prevent the irreversible consequences of the oxidative damage induced by SOD1^G93A^ mutation. Although not fully defined by the present data, the mechanisms underlying this selective endurance should entail the ER–mitochondria connections and the configuration of MAMs, whose role in cellular redox control has been already documented in different cell models [42]. In agreement with this hypothesis, the MAM constituent Mfn2 showed an increase in SC astrocytes as opposed to a decrease in MC ones, corroborating the hypothesis of a differential ER involvement in the cellular redox control of these two districts.

### 4.3. Limitations

The experimental nature of our study and its focus on SOD1^G93A^ mice obviously implies the need for further investigations to verify whether the divergent endurance against the redox stress also characterizes the clinical reality of ALS. However, our choice selected one of the most widely used experimental models of this disease. Its similarities with both familial and sporadic clinical ALS have already been reported. In agreement with this notion, the divergent FDG uptake in MC and SC astrocytes harvested in newborn pups indicates that the redox damage is heterogeneously distributed within the central nervous system soon after birth and thus far before the onset of motor impairment.

In the present study, we only evaluated astrocytes from mutated mice, while the interference by or to motor neurons was not investigated. Several studies documented that the motor impairment occurs in SOD1^G93A^ mice only when the mutation involves both astrocytes and motor neurons. Accordingly, the present data do not elucidate the molecular mechanisms underlying the reciprocal damage between these two cell populations. Nevertheless, our focus was to define whether the link between FDG uptake and metabolic response to redox stress characterizes the early phases of motor impairment and is not limited to the symptomatic ALS phases as reported both in patients and SOD1^G93A^ mice.

## 5. Conclusions

The present data indicate that the irreversible oxidative damage associated with the SOD1^G93A^ mutation is not counterbalanced by the activation of cytosolic PPP. By contrast, the activation of the ER-PPP provides a relative protection despite the marked alteration of this organelle that characterizes the studied experimental models as well as the pathological specimens from ALS patients. The signals able to selectively activate this pathway within the SC astrocytes remain to be elucidated. However, their comprehension might configure a new target to understand the mechanisms underlying ALS progression.

## Data Availability

The data presented in this study are available on request from the corresponding author. The data are not publicly available due to the contribution of many different labs.

## References

[1] Dewil M., Van Den Bosch L., Robberecht W. (2007). Microglia in amyotrophic lateral sclerosis. Acta Neurol. Belg..

[2] Ferraiuolo L., Meyer K., Sherwood T.W., Vick J., Likhite S., Frakes A., Miranda C.J., Braun L., Heath P.R., Pineda R. (2016). Oligodendrocytes contribute to motor neuron death in ALS via SOD1-dependent mechanism. Proc. Natl. Acad. Sci. USA.

[3] Bowling A.C., Beal M.F. (1995). Bioenergetic and oxidative stress in neurodegenerative diseases. Life Sci..

[4] Singh A., Kukreti R., Saso L., Kukreti S. (2019). Oxidative Stress: A Key Modulator in Neurodegenerative Diseases. Molecules.

[5] Mizielinska S., Lashley T., Norona F.E., Clayton E., Ridler C.E., Fratta P., Isaacs A.M. (2013). C9orf72 frontotemporal lobar degeneration is characterised by frequent neuronal sense and antisense RNA foci. Acta Neuropathol..

[6] Forsberg K., Andersen P.M., Marklund S.L., Brannstrom T. (2011). Glial nuclear aggregates of superoxide dismutase-1 are regularly present in patients with amyotrophic lateral sclerosis. Acta Neuropathol..

[7] Miller D.W., Cookson M.R., Dickson D.W. (2004). Glial cell inclusions and the pathogenesis of neurodegenerative diseases. Neuron Glia Biol..

[8] Bruijn L.I., Becher M.W., Lee M.K., Anderson K.L., Jenkins N.A., Copeland N.G., Sisodia S.S., Rothstein J.D., Borchelt D.R., Price D.L. (1997). ALS-Linked SOD1 Mutant G85R Mediates Damage to Astrocytes and Promotes Rapidly Progressive Disease with SOD1-Containing Inclusions. Neuron.

[9] Sambuceti G., Cossu V., Bauckneht M., Morbelli S., Orengo A., Carta S., Ravera S., Bruno S., Marini C. (2021). 18F-fluoro-2-deoxy-d-glucose (FDG) uptake. What are we looking at?. Eur. J. Nucl. Med. Mol. Imaging.

[10] Bánhegyi G., Benedetti A., Fulceri R., Senesi S. (2004). Cooperativity between 11beta-Hydroxysteroid Dehydrogenase Type 1 and Hexose-6-phosphate Dehydrogenase in the Lumen of the Endoplasmic Reticulum. J. Biol. Chem..

[11] Csala M., Bánhegyi G., Benedetti A. (2006). Endoplasmic reticulum: A metabolic compartment. FEBS Lett..

[12] Marini C., Ravera S., Buschiazzo A., Bianchi G., Orengo A.M., Bruno S., Bottoni G., Emionite L., Pastorino F., Monteverde E. (2016). Discovery of a novel glucose metabolism in cancer: The role of endoplasmic reticulum beyond glycolysis and pentose phosphate shunt. Sci. Rep..

[13] Cossu V., Bauckneht M., Bruno S., Orengo A.M., Emionite L., Balza E., Castellani P., Piccioli P., Miceli A., Raffa S. (2020). The Elusive Link Between Cancer FDG Uptake and Glycolytic Flux Explains the Preserved Diagnostic Accuracy of PET/CT in Diabetes. Transl. Oncol..

[14] Cossu V., Bonanomi M., Bauckneht M., Ravera S., Righi N., Miceli A., Morbelli S., Orengo A.M., Piccioli P., Bruno S. (2020). Two high-rate pentose-phosphate pathways in cancer cells. Sci. Rep..

[15] Bauckneht M., Cossu V., Castellani P., Piccioli P., Orengo A.M., Emionite L., Di Giulio F., Donegani M.I., Miceli A., Raffa S. (2020). FDG uptake tracks the oxidative damage in diabetic skeletal muscle: An experimental study. Mol. Metab..

[16] Marini C., Cossu V., Bonifacino T., Bauckneht M., Torazza C., Bruno S., Castellani P., Ravera S., Milanese M., Venturi C. (2020). Mechanisms underlying the predictive power of high skeletal muscle uptake of FDG in amyotrophic lateral sclerosis. EJNMMI Res..

[17] Bauckneht M., Pastorino F., Castellani P., Cossu V., Orengo A.M., Piccioli P., Emionite L., Capitanio S., Yosifov N., Bruno S. (2020). Increased myocardial 18F-FDG uptake as a marker of Doxorubicin-induced oxidative stress. J. Nucl. Cardiol..

[18] Cossu V., Marini C., Piccioli P., Rocchi A., Bruno S., Orengo A.M., Emionite L., Bauckneht M., Grillo F., Capitanio S. (2019). Obligatory role of endoplasmic reticulum in brain FDG uptake. Eur. J. Nucl. Med. Mol. Imaging.

[19] Marini C., Cistaro A., Campi C., Calvo A., Caponnetto C., Nobili F., Fania P., Beltrametti M.C., Moglia C., Novi G. (2016). A PET/CT approach to spinal cord metabolism in amyotrophic lateral sclerosis. Eur. J. Nucl. Med. Mol. Imaging.

[20] Marini C., Morbelli S., Cistaro A., Campi C., Caponnetto C., Bauckneht M., Bellini A., Buschiazzo A., Calamia I., Beltrametti M.C. (2018). Interplay between spinal cord and cerebral cortex metabolism in amyotrophic lateral sclerosis. Brain.

[21] Gurney M.E., Pu H., Chiu A.Y., Dal Canto M.C., Polchow C.Y., Alexander D.D., Caliendo J., Hentati A., Kwon Y.W., Deng H.X. (1994). Motor neuron degeneration in mice that express a human Cu,Zn superoxide dismutase mutation. Science.

[22] Kim R.B., Irvin C.W., Tilva K.R., Mitchell C.S. (2015). State of the field: An informatics-based systematic review of the SOD1-G93A amyotrophic lateral sclerosis transgenic mouse model. Amyotroph. Lateral Scler. Front. Degener..

[23] Bonifacino T., Cattaneo L., Gallia E., Puliti A., Melone M., Provenzano F., Bossi S., Musante I., Usai C., Conti F. (2017). In-vivo effects of knocking-down metabotropic glutamate receptor 5 in the SOD1 G93A mouse model of amyotrophic lateral sclerosis. Neuropharmacology.

[24] Paluzzi S., Alloisio S., Zappettini S., Milanese M., Raiteri L., Nobile M., Bonanno G. (2007). Adult astroglia is competent for Na^+^/Ca^2+^ exchanger-operated exocytotic glutamate release triggered by mild depolarization. J. Neurochem..

[25] Miceli A., Cossu V., Marini C., Castellani P., Raffa S., Donegani M.I., Bruno S., Ravera S., Emionite L., Orengo A.M. (2020). 18F-Fluorodeoxyglucose Positron Emission Tomography Tracks the Heterogeneous Brain Susceptibility to the Hyperglycemia-Related Redox Stress. Int. J. Mol. Sci..

[26] Kruger N.J. (1994). The Bradford Method for Protein Quantitation. Methods Mol. Biol..

[27] Scussolini M., Bauckneht M., Cossu V., Bruno S., Orengo A.M., Piccioli P., Capitanio S., Yosifov N., Ravera S., Morbelli S. (2019). G6Pase location in the endoplasmic reticulum: Implications on compartmental analysis of FDG uptake in cancer cells. Sci. Rep..

[28] Naon D., Zaninello M., Giacomello M., Varanita T., Grespi F., Lakshminaranayan S., Serafini A., Semenzato M., Herkenne S., Hernández-Alvarez M.I. (2016). Critical reappraisal confirms that Mitofusin 2 is an endoplasmic reticulum–mitochondria tether. Proc. Natl. Acad. Sci. USA.

[29] Csordás G., Renken C., Várnai P., Walter L., Weaver D., Buttle K.F., Balla T., Mannella C.A., Hajnóczky G. (2006). Structural and functional features and significance of the physical linkage between ER and mitochondria. J. Cell Biol..

[30] Kantzer C.G., Boutin C., Herzig I.D., Wittwer C., Reiß S., Tiveron M.C., Drewes J., Rockel T.D., Ohlig S., Ninkovic J. (2017). Anti-ACSA-2 defines a novel monoclonal antibody for prospective isolation of living neonatal and adult astrocytes. Glia.

[31] Bennett M.L., Bennett F.C., Liddelow S.A., Ajami B., Zamanian J.L., Fernhoff N.B., Mulinyawe S.B., Bohlen C.J., Adil A., Tucker A. (2016). New tools for studying microglia in the mouse and human CNS. Proc. Natl. Acad. Sci. USA.

[32] Riganti C., Gazzano E., Polimeni M., Aldieri E., Ghigo D. (2012). The pentose phosphate pathway: An antioxidant defense and a crossroad in tumor cell fate. Free. Radic. Biol. Med..

[33] Yang H.C., Stern A., Chiu D.T. (2020). G6PD: A hub for metabolic reprogramming and redox signaling in cancer. Biomed. J..

[34] Szymański J., Janikiewicz J., Michalska B., Patalas-Krawczyk P., Perrone M., Ziółkowski W., Duszyński J., Pinton P., Dobrzyń A., Więckowski M.R. (2017). Interaction of Mitochondria with the Endoplasmic Reticulum and Plasma Membrane in Calcium Homeostasis, Lipid Trafficking and Mitochondrial Structure. Int. J. Mol. Sci..

[35] Xia M., Zhang Y., Jin K., Lu Z., Zeng Z., Xiong W. (2019). Communication between mitochondria and other organelles: A brand-new perspective on mitochondria in cancer. Cell Biosci..

[36] Giorgi C., De Stefani D., Bononi A., Rizzuto R., Pinton P. (2009). Structural and functional link between the mitochondrial network and the endoplasmic reticulum. Int. J. Biochem. Cell Biol..

[37] Sokoloff L., Reivich M., Kennedy C., Des Rosiers M.H., Patlak C.S., Pettigrew K.D., Sakurada O., Shinohara M. (1977). The [14C]deoxyglucose method for the measurement of local cerebral glucose utilization: Theory, procedure, and normal values in the conscious and anesthetized albino rat. J. Neurochem..

[38] Bachelard H.S. (1971). Specificity and kinetic properties of monosaccharide uptake into guinea pig cerebral cortex in vitro. J. Neurochem..

[39] Caracó C., Aloj L., Chen L.-Y., Chou J.Y., Eckelman W.C. (2000). Cellular Release of [18F]2-Fluoro-2-deoxyglucose as a Function of the Glucose-6-phosphatase Enzyme System. J. Biol. Chem..

[40] Bublitz C., Steavenson S. (1988). The pentose phosphate pathway in the endoplasmic reticulum. J. Biol. Chem..

[41] Sullivan P.G., Rabchevsky A.G., Keller J.N., Lovell M., Sodhi A., Hart R.P., Scheff S.W. (2004). Intrinsic differences in brain and spinal cord mitochondria: Implication for therapeutic interventions. J. Comp. Neurol..

[42] Van Vliet A.R., Verfaillie T., Agostinis P. (2014). New functions of mitochondria associated membranes in cellular signaling. Biochim. Biophys. Acta BBA Mol. Cell Res..

